# Outcome of follicular lymphoma grade 3: is anthracycline necessary as front-line therapy?

**DOI:** 10.1038/sj.bjc.6601006

**Published:** 2003-07-01

**Authors:** I Chau, R Jones, D Cunningham, A Wotherspoon, N Maisey, A R Norman, P Jain, L Bishop, A Horwich, D Catovsky

**Affiliations:** 1 Department of Medicine, Royal Marsden Hospital, Downs Road, Sutton, Surrey SM2 5PT, UK; 2 Department of Histopathology, Royal Marsden Hospital, Downs Road, Sutton, Surrey SM2 5PT, UK; 3 Department of Computing, Royal Marsden Hospital, Downs Road, Sutton, Surrey SM2 5PT, UK; 4 Academic Department of Haematology, Royal Marsden Hospital, Fulham Road, London, SW3 6JJ, UK; 5 Department of Academic Radiotherapy, Royal Marsden Hospital, Downs Road, Sutton, Surrey, SM2 5PT, UK

**Keywords:** follicular lymphoma, WHO classification, cytological grading, anthracycline

## Abstract

A grading system (grades 1–3) for follicular lymphoma (FL) is used in the WHO classification for lymphoid malignancies based on the absolute number of centroblasts in the neoplastic follicles. Grade 3 FL is further subdivided into 3a and 3b depending on the presence or absence of centrocytes. A total of 231 patients with FL, referred from 1970 to 2001, were identified from our prospectively maintained database. Original diagnostic materials were available for review on 215 patients and these were reclassified according to the WHO grading system. Follicular lymphoma grades 1, 2 and 3 accounted for 92, 68 and 55 patients, respectively. No significant overall survival (OS) differences were observed among FL grades 1–3 (log rank *P*=0.25) or between grades 3a and 3b (log rank *P*=0.20). No significant failure-free survival (FFS) differences were observed among FL grades 1–3 (log rank *P*=0.72) or between grades 3a and 3b (log rank *P*=0.11). First-line anthracyclines did not influence OS or FFS (log rank *P*=0.86, *P*=0.58, respectively) in patients with FL grade 3. There are long-term survivors among patients with FL grade 3 with a continuing risk of relapse. Anthracyclines did not appear to influence survival or disease relapses when given as front-line therapy in our series. The role of anthracyclines should be further evaluated in large randomised studies.

Non-Hodgkin's lymphoma (NHL) is increasing in incidence with more than 287 000 cases diagnosed worldwide each year (Ferlay *et al*, 2001). Follicular lymphoma (FL) is the second most frequent type of NHL comprising 22% of all NHL (Armitage and Weisenburger, 1998). Follicular large cell lymphoma (FLCL) is an uncommon subtype of FL accounting for 10–40% of FL (Newell *et al*, 1987; Martin *et al*, 1995; Miller *et al*, 1997; Rodriguez *et al*, 1999) and only 2–4% of all NHLs (Rosenberg *et al*, 1982; Newell *et al*, 1987). In the US, the incidence rate for FLCL was 0.5 per 100 000 in 1998 (Ries *et al*, 2001).

Over the last two decades, several classifications have been proposed for NHL. In the Working Formulation (Rosenberg *et al*, 1982), FLCL is diagnosed when the majority of neoplastic cells within the follicles are large cleaved or noncleaved cells, although large noncleaved cells usually predominate. However, the exact proportion of large cells required to make a diagnosis of FLCL was not specified. In the Revised European-American Lymphoma (REAL) classification, a grading system (grades I–III) was introduced in FL (Harris *et al*, 1994), but no specific recommendations for grading criteria were given.

The recently introduced World Health Organisation (WHO) classification (Nathwani *et al*, 2001) recommends a similar three-grade system (grades 1–3) based on counting the absolute number of centroblasts in 10 neoplastic follicles, expressed per high-power microscopic field (h.p.f.) of 0.159 mm^2^. Grade 1 cases have 0–5 centroblasts per h.p.f., grade 2 cases have 6–15 centroblasts per h.p.f. and grade 3 cases have >15 centroblasts per h.p.f. This method of histological grading has been shown to predict both overall survival (OS) and failure-free survival (FFS) (Martin *et al*, 1995). Furthermore, grade 3 FL can be subdivided for investigational purposes according to the number of centroblasts. In grade 3a, there are >15 centroblasts per h.p.f., but centrocytes are still present, while grade 3b has solid sheets of centroblasts with no centrocytes.

Due to the imprecise method of diagnosing FLCL, relative infrequency of the disease, small and retrospective nature and short follow-up of many published studies, there is still controversy among investigators with regard to its behaviour. Most importantly, there is no agreement of whether FLCL is curable, especially when treated with aggressive doxorubicin-containing regimens. The grading method by Mann and Berard (1983) used in the WHO classification allows more uniform reporting of the FL subtypes; the cohort of patients being studied can therefore be better defined.

Here we report on the long-term follow-up of 215 patients with FL classified according to the Mann & Berard/WHO grading system focusing on patients with FL grade 3. The objectives in this report are: (1) to compare survival of patients with FL grade 3 with grades 1 and 2, (2) to compare survival of patients with FL grades 3a and 3b, (3) to determine the curability of FL grade 3 with standard therapy and (4) to compare survival of patients with FL grade 3 treated with anthracycline or anthracenedione (mitoxantrone)-based chemotherapy *vs* other regimens.

## PATIENTS AND METHOD

Between 1970 and 2001, 231 patients with FL were evaluated at the Royal Marsden Hospital. Medical details, laboratory analyses, treatment details including previous treatment before presenting to our institution and treatment outcome were recorded in a prospectively maintained database. Original diagnostic pathological materials obtained at presentation were independently reviewed by two haematopathologists (AW and PJ) in September 2001 and classified according to the WHO grading system. Discrepancies were resolved following discussion over a double-headed microscope and review of centroblast counts. In all, 16 patients were excluded because of missing diagnostic material. A total of 215 patients formed the cohort in our study of whom 92 had grade 1, 68 had grade 2 and 55 had grade 3 FL (44 had grade 3a and 11 had grade 3b).

### Treatment details

Table 1

**Table 1:** Initial management of patients with follicular lymphoma grade 3

**Treatment**	**Number (total *n*=55)**
*Anthracycline-based chemotherapy*	*n*=15
CHOP (+RT)	8 (+1)
FMD	1
FAD	3
MACOP-B	1
PMitCEBO	1
	
*Nonanthracycline-based chemotherapy*	*n*=21
Chlorambucil/prednisolone	3
Chlorambucil	6
COP/CVP	7
CVP+rituximab	3
Fludarabine	1
LOP	1
	
*Radiotherapy*	*n*=12
Involved field	9
Extended field	3
	
*Surveillance*	*n*=7

CHOP=cyclophosphamide, doxorubicin, vincristine and prednisolone; RT=radiotherapy; FMD=fludarabine, mitoxantrone, dexamethasone; FAD=fludarabine, doxorubicin, dexamethasone; MACOP-B=methotrexate, doxorubicin, cyclophosphamide, vincristine, prednisolone and bleomycin; PMitCEBO=prednisolone, mitoxantrone, cyclophosphamide, etoposide, bleomycin and vincristine; COP/CVP=cyclophosphamide, vincristine and prednisolone. Drugs are given in different doses and schedules in COP and CVP; LOP=lomustine, vincristine and prednisolone.

shows the details of initial management of the 55 patients with grade 3 FL. In all, 10 patients had received prior treatment before presenting to our institution. Seven patients were managed with deferred therapy initially, five of these patients eventually required treatment after a median of 21 months (range: 5–122 months). Two patients are still on surveillance only. During follow-up, patients received a median of two lines of treatment (range: 0–9) in total. For the purpose of analysis, both doxorubicin and mitoxantrone were considered as anthracyclines. In all, 14 patients received anthracycline-based combination as subsequent treatment including six who received their first anthracycline-based chemotherapy after transformation into diffuse large cell histology. In total, 25 patients did not receive any anthracycline, including seven who received radiotherapy (RT) alone.

### Statistical analysis

Baseline characteristics among three grades of FL were compared using the *χ*^2^ test for categorical variables (gender, stage, systemic symptoms and performance status). Continuous variables (serum lactate dehydrogenase (LDH) and age) were tested for normality using the Kolmogorov–Smirnov test. Analysis of variance (ANOVA) was used for continuous variables with normal distribution (age) and Kruskal–Wallis test was used for continuous variables with non-normal distribution (LDH).

Overall survival was calculated from the date of diagnosis until death from any cause or last follow-up. Failure-free survival was calculated from the date of diagnosis until disease progression, relapse, death from any cause or last follow-up evaluation. Cause-specific survival (CSS) was calculated from the date of diagnosis until death due to disease or treatment toxicity. Overall survival, FFS and CSS were estimated using the Kaplan–Meier method (Kaplan and Meier, 1958). Comparisons of survival curves were performed using the log-rank test (Peto and Peto, 1972).

Univariate analysis was performed in patients with FL grade 3 using forward stepwise Cox proportional hazards regression modelling (Cox and Oakes, 1984). Age, performance status, LDH, haemoglobin, platelet count, white blood cell and lymphocyte count, albumin, alkaline phosphatase, erythrocyte sedimentation rate (ESR) and *β*2 microglobulin were used as continuous variables. Gender, stage of disease (I+II *vs* III+IV), systemic symptoms (presence *vs* absence), bulky disease over 5 cm (presence *vs* absence), marrow involvement (presence *vs* absence) and first-line use of anthracycline-based chemotherapy (yes *vs* no) were used as categorical variables. Data were analysed using the SPSS package version 10.1.4 (SPSS Inc. Chicago, IL, USA) in January 2002.

## RESULTS

### Clinical features

Table 2

**Table 2:** Patient characteristics

	**Number of patients (%)**			
**Clinical features**	**Grade 1**	**Grade 2**	**Grade 3**	***P*-value**
Total number of patients	92	68	55	
Median age (years) (range)	49 (28–87)	54.5 (34–78)	53 (18–81)	0.57
				
*Gender*				
Male	38	31	29	0.40
Female	54	37	26	
				
*Stage at presentation*				
I	9 (9.8)	12 (17.6)	13 (23.6)	
II	7 (7.6)	11 (16.2)	6 (10.9)	0.09
III	25 (27.2)	20 (29.4)	15 (27.3)	
IV	51 (55.4)	25 (36.8)	21 (38.2)	
				
*Systemic symptoms*				
Absent	68 (73.9)	52 (76.5)	42 (76.4)	0.92
Present	24 (26.1)	16 (23.5)	13 (23.6)	
				
*Performance status*				
0–1	53 (57.6)	36 (52.9)	39 (70.9)	
2–4	1 (1.1)	5 (7.4)	4 (7.3)	0.62
Unknown	38 (41.3)	27 (39.7)	12 (21.8)	(0.129)^a^
				
*LDH*				
Normal	39 (42.4)	31 (45.6)	20 (36.3)	
Elevated	19 (20.7)	12 (17.6)	10 (18.2)	0.72^b^
Unknown	34 (37.0)	25 (36.8)	25 (45.5)	

LDH=lactate dehydrogenase.

a If patients with unknown performance status are excluded.

b Using Kruskal–Wallis test.

shows the clinical features of patients with all three grades of FL. No statistical differences in baseline clinical features were observed among them. Nine patients (16%) with FL grade 3 had subsequent transformation to diffuse large cell histology during follow-up.

### Outcome

**Figure 1:**
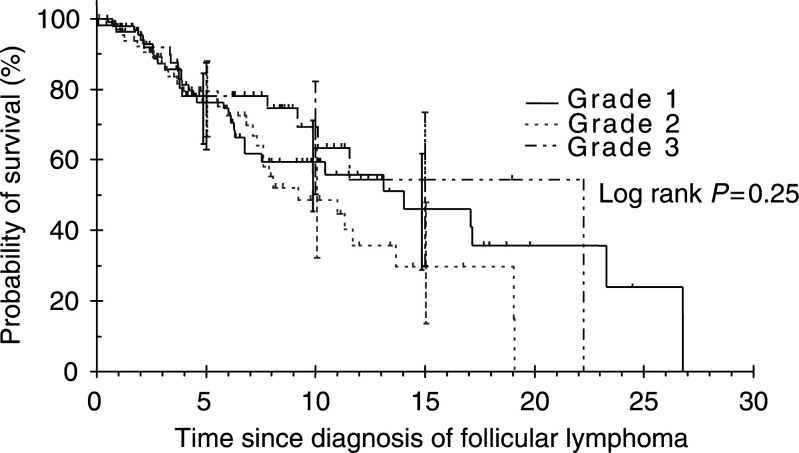
Overall survival of follicular lymphoma by grade (years).

**Figure 2:**
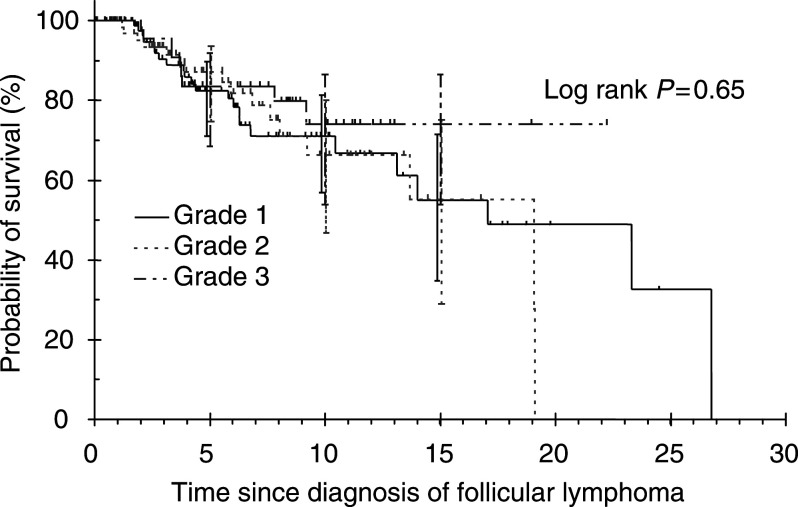
Cause-specific survival of follicular lymphoma by grade (years).

**Figure 3:**
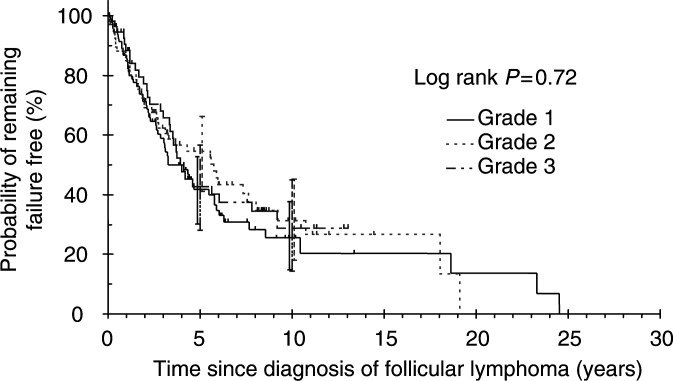
Failure-free survival of follicular lymphoma by grade.

The median follow-up for grades 1, 2 and 3 was 54.7, 57.2 and 79.6 months, respectively. No survival differences were observed among grades 1–3 (log rank *P*=0.25, Figure 1

). The median survival for grades 1, 2 and 3 was 14, 9.2 and 22.2 years, respectively. Overall survival rates at 10 years were 59.5% (95% confidence interval (CI)=45.4–71.1%), 48.7% (95% CI=32.2–63.3%) and 69.2% (95% CI=50.2–82.2%) for grades 1, 2 and 3, respectively. No CSS differences in grades 1–3 were observed (log rank *P*=0.65, Figure 2

). Cause-specific survival rates at 10 years were 71.1% (95% CI=57–81.4%), 66.3% (95% CI=46.8–80%) and 74.1% (95% CI=53.9–86.5%) for grades 1, 2 and 3, respectively. No FFS differences were observed among grades 1, 2 and 3 (log rank *P*=0.72, Figure 3

). Failure-free survival rates at 10 years were 25.5% (95% CI=14.9–37.6%), 31.2% (95% CI=18.0–45.3%) and 28.8% (95% CI=14.4–44.9%) for grades 1–3, respectively.

**Figure 4:**
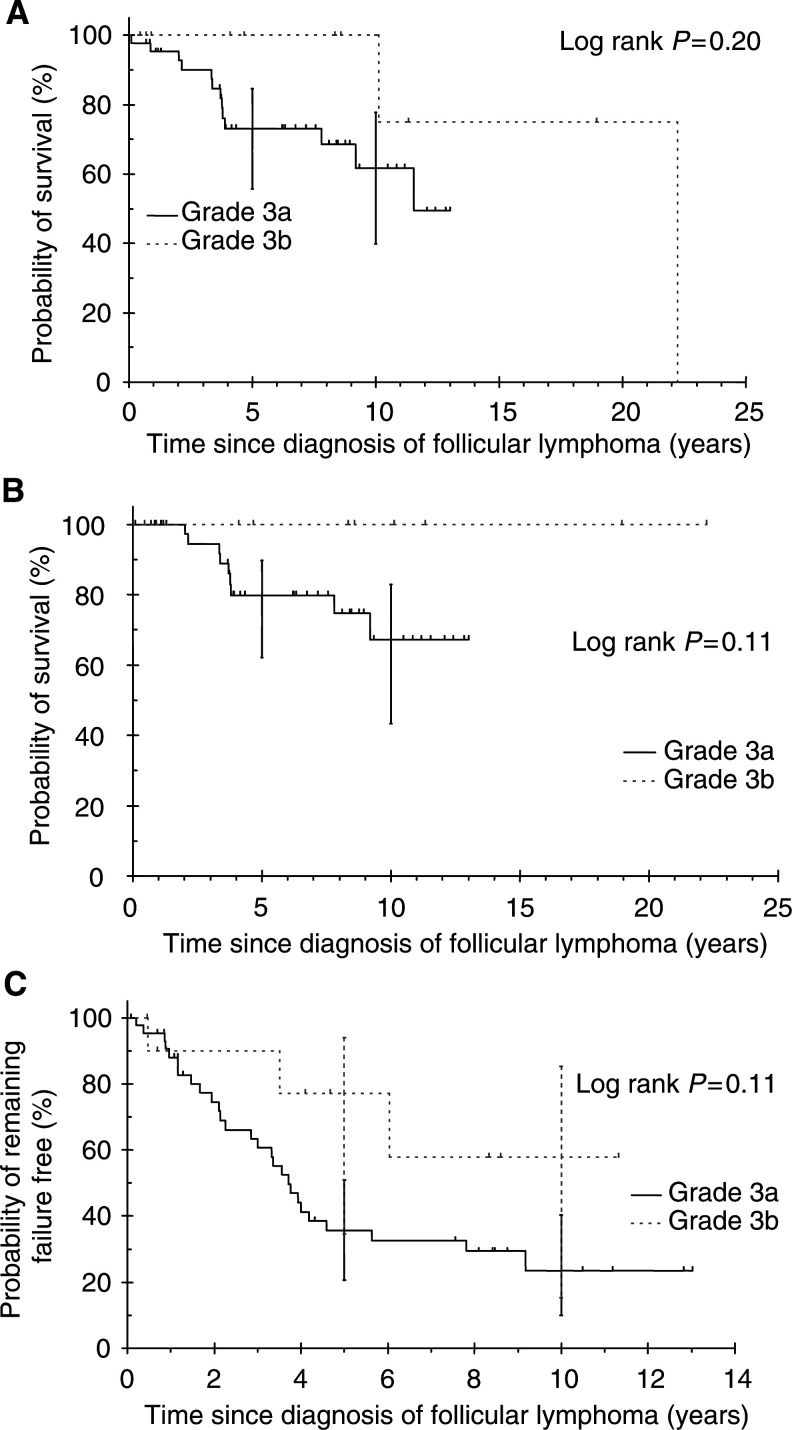
Survival for grade 3a and 3b follicular lymphoma: (**A**) overall survival; (**B**) cause-specific survival; (**C**) failure-free survival.

Restricting the analysis to FL grades 3a and 3b, the median follow-up was 82.2 and 56.9, months, respectively. No overall survival difference was observed between 3a and 3b (log rank *P*=0.20, Figure 4A

). The median survival was 11.5 and 22.2 years for grades 3a and 3b, respectively. Overall survival rates at 10 years were 61.7% (95% CI=39.8–77.7%) and 100% (not possible to calculate the 95% CI as no events have occurred) for grades 3a and 3b, respectively. No CSS difference was observed between 3a and 3b (log rank *P*=0.11, Figure 4B). Cause-specific survival rates at 10 years were 67.3% (95% CI=43.4–82.9%) and 100% for grades 3a and 3b, respectively. No FFS difference was observed between grades 3a and 3b (log rank *P*=0.11, Figure 4C). Failure-free survival rates at 10 years were 23.5% (95% CI=10–40.3%) and 57.9% (95% CI=15.3–85.2%) for grades 3a and 3b, respectively.

A total of 40 patients with FL grade 3 are alive. Among them, 18 patients are in continuous remission after first therapy. Of the 15 patients who died, nine died of lymphoma. Six died from unrelated causes including two from second primary malignancy, two from infection, one from chronic renal failure and one from pulmonary embolism. No patients died of cardiovascular disease. Both fatalities in patients with FL grade 3b resulted from colorectal cancer 22 and 10 years after the initial diagnosis of follicular lymphoma.

**Figure 5:**
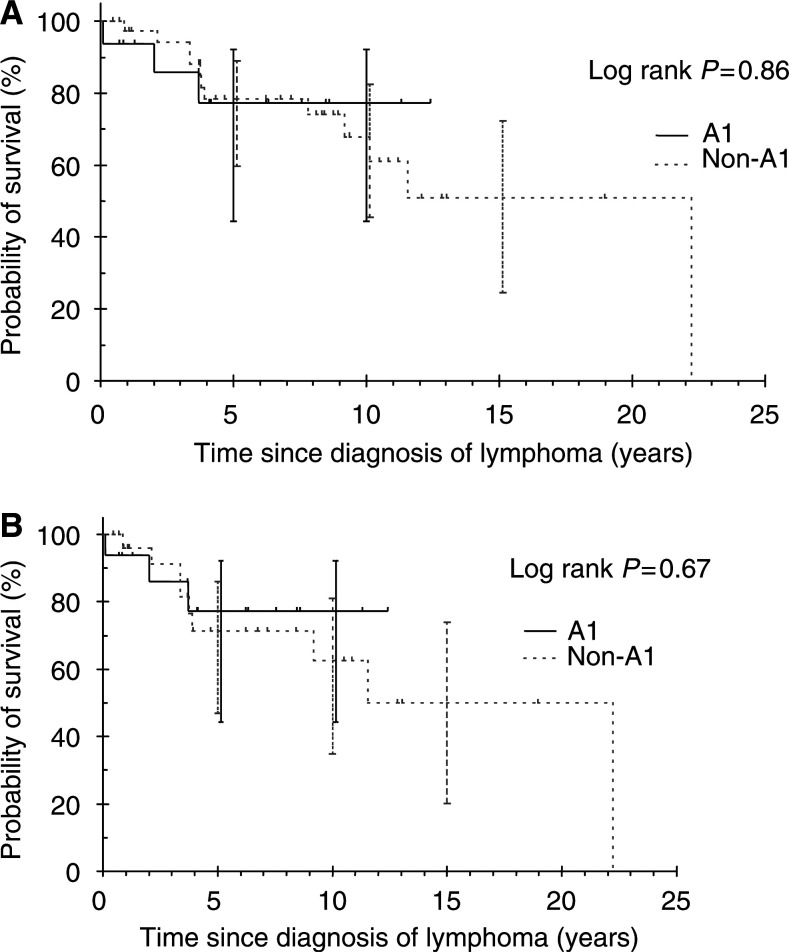
Survival for patients with grade 3 follicular lymphoma receiving anthracyclines. (**A**) Overall survival for all patients with grade 3 follicular lymphoma. A1 group includes patients who received anthracyclines as their first-line treatment. Non-A1 group includes patients who did not receive anthracyclines as their first-line treatment. (**B**) Overall survival for patients with grade 3 follicular lymphoma excluding those with limited disease and received radiotherapy only as initial treatment.

No survival difference was seen between patients with FL grade 3 who received anthracycline-based chemotherapy as first-line therapy (the A1 group) and those who did not (the non-A1 group) (log rank *P*=0.86, Figure 5A

). However, no deaths were observed after 4 years in the A1 group. Even when patients with limited stage disease who received RT only as first-line therapy were excluded, no survival difference was seen between the A1 group and the non-A1 group (log rank *P*=0.67, Figure 5B).

**Figure 6:**
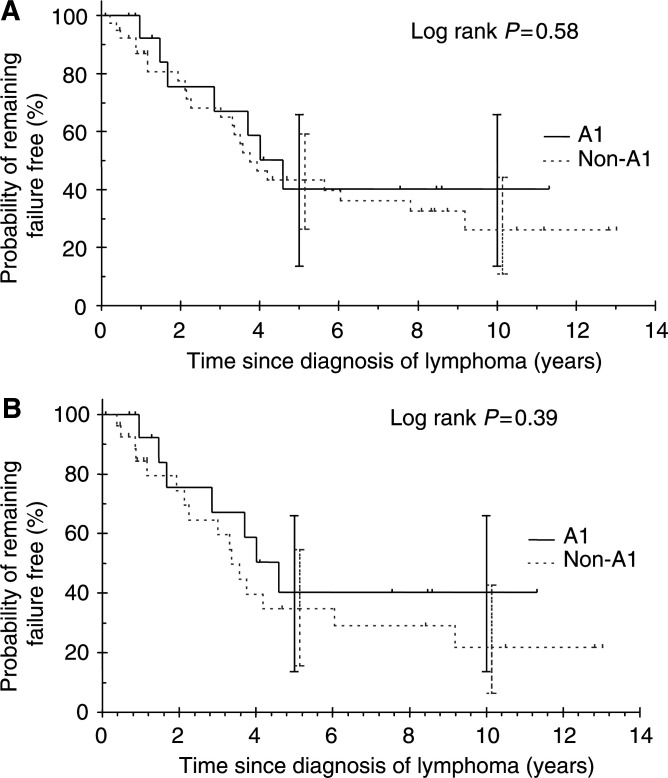
Failure-free survival for patients with grade 3 follicular lymphoma receiving anthracyclines: (**A**) for all patients with grade 3 follicular lymphoma and (**B**) for patients with grade 3 follicular lymphoma excluding those with limited disease and received radiotherapy only as initial treatment.

No FFS difference was seen between the A1 group and the non-A1 group (log rank *P*=0.58, Figure 6A

). However, no relapses were observed after 5 years in the A1 group. Even when patients with limited stage disease who received RT only as first-line therapy were excluded, no FFS difference was seen between the A1 and the non-A1 groups (log rank *P*=0.39, Figure 6B).

In order to investigate whether anthracycline-based chemotherapy may be more beneficial in either grade 3a or 3b, a further analysis was performed. Patients with FL grade 3a or 3b were divided according to whether they received anthracycline or nonanthracycline-based regimen as their first chemotherapy regimen. This therefore included patients who initially received RT but experienced disease relapse or progression, and patients who were initially on surveillance but required chemotherapy. In patients with FL grade 3a, a similar proportion of patients experienced disease relapse or progression whether they received anthracycline or nonanthracycline-based chemotherapy (*P*=0.76). However, in patients with FL grade 3b and who had received anthracycline-based chemotherapy, none experienced disease relapse or progression (Table 3

**Table 3:** Patients with follicular lymphoma grade 3 receiving anthracycline as first chemotherapy regimen including those who relapsed after initial radiotherapy and those who progressed during initial surveillance

	**Number of patients**	
	**Grade 3a**	**Grade 3b**
First-line anthracycline-based chemotherapy	13	4
Disease relapse/progression	9	0
First-line nonanthracycline-based chemotherapy	23	5
Disease relapse/progression	17	2

).

### Factors predicting survival

Univariate analysis was performed to identify predictive factors for survival in patients with FL grade 3 (Table 4

**Table 4:** Univariate analysis of patients with follicular lymphoma grade 3

		**95% Confidence interval**		
**Factors**	**Hazard ratio**	**Lower limit**	**Upper limit**	***P*-value**
Age	1.036	0.995	1.08	0.085
Performance status	2.239	1.018	4.924	0.045
LDH	1.003	1.000	1.006	0.086
*β*_2_-microglobulin	8.205	1.528	44.063	0.014
Stage III–IV	3.944	0.879	17.701	0.073
Serum albumin	0.661	0.463	0.943	0.022
First-line treatment with anthracyclines	0.89	0.246	3.218	0.86

). Increasing age, poor performance status, elevated LDH, elevated *β*2 microglobulin and stage III–IV disease were found to be significant or borderline significant poor prognostic factors. Normal or elevated level of albumin was a favourable prognostic factor. First-line treatment with anthracycline-based chemotherapy was not found to be significant. However, multivariate analysis was not performed because first only 15 events have occurred in patients with FL grade 3, second 10 patients had prior treatment before presenting to our institution and third, some of the baseline laboratory analyses were not available.

## DISCUSSION

The natural history of FL is often considered as having an indolent growth pattern, but remains essentially incurable when disseminated. Because of the inconsistent histological criteria for making the diagnosis of FLCL, many of the reported series may not be directly comparable. Nevertheless, it emerges from the published literature that, unlike other subtypes of FL, a small group of patients is free of disease with prolonged follow-up. The Mann & Berard grading system adopted by the WHO provides a more uniform system of diagnosing different subtypes of FL, therefore allowing a more consistent approach in defining the patient cohort.

In our first objective to compare survival of patients with FL grade 3 with grades 1 and 2, we found no significant differences among the histological grades. Two series in the literature reported contradictory results (Martin *et al*, 1995; Miller *et al*, 1997). In the Nebraska study, 64 patients with FLCL diagnosed using the Mann & Berard method had worse survival compared to the combined group of 42 patients with follicular small cell and follicular mixed cell lymphoma patients (*P*=0.0035). In contrast, Miller *et al* (1997) reported the long-term outcome of 389 patients registered on SWOG studies. No differences in OS among the three follicular histological subgroups were found (*P*=0.40). Our 10-year survival of 69.2% in patients with FL grade 3 compared well with other series (Table 5

**Table 5:** Survival of patients with follicular large cell lymphoma in recently reported series

**Studies**	**5-year overall survival (%)**	**10-year overall survival (%)**
Our study (2002)^a^	78 (95% CI: 63–88%)	69 (95% CI: 50–82%)
Nebraska study (Anderson *et al*, 1993)	45	NS
Stanford study (Bartlett *et al*, 1994)	75	54
GELA (Wendum *et al*, 1997)	58	NS
SWOG study (Miller *et al*, 1997)	NS	37
Nebraska study (Tezcan *et al*, 1999) stage I–II	NS	44
MD Anderson (Rodriguez *et al*, 1999)	72	NS
MD Anderson (Rodriguez *et al*, 2000)	NS	31

CI=confidence interval. NS=not stated in publications.

a Patients with follicular lymphoma grade 3.

). Although our inclusion of stage I and II disease patients may explain the better survival, our long-term outcome still compares favourably with series which included stage I and II only (Tezcan *et al*, 1999).

Although there is no difference in CSS among grades 1, 2 and 3, no disease-related deaths occurred beyond 10 years for grade 3. This suggests that a proportion of patients may be potentially curable or successfully salvaged, but prolonged survival means that patients may die from unrelated causes. A long-term follow-up of 62 patients from the MD Anderson series treated between 1973 and 1981 showed that lymphoma-related mortality continued to occur beyond 15 years (Rodriguez *et al*, 2000) suggesting that longer follow-up for our cohort of patients is necessary before this plateau in CSS can be confirmed. No difference in FFS was observed among grades 1–3. Patients with FL grade 3 had a continuing risk of relapses with 10 patients relapsing after 5 years. Although no relapses have been observed beyond 9 years, only five patients are failure free at this time point. Again longer follow-up and larger patient number are necessary to determine the curability of grade 3.

Our study provides an opportunity to examine the difference in behaviour between grades 3a and 3b. There does not appear to be any survival differences between grades 3a and 3b. However, the small number of patients with grade 3b in this study means only 10-year survival differences in excess of 30% between grades 3a and 3b could be detected with at least 80% power. Nevertheless, it is interesting that no patients with FL grade 3b died of lymphoma and both deaths in patients with FL grade 3b resulted from second primary colorectal cancers. No difference in FFS was, however, evident between grades 3a and 3b with late relapses after 5 years occurring in both groups. No definitive curability could be seen yet with either grade 3a or grade 3b.

The Working Formulation classified FLCL into an intermediate grade NHL together with diffuse large B cell non-Hodgkin's lymphoma (DLBCL). As a result, researchers often treated these patients with intensive anthracycline-containing chemotherapy similar to those used in DLBCL. The majority of the patients in the recently reported series have been treated with anthracyclines (Anderson *et al*, 1993; Bartlett *et al*, 1994; Miller *et al*, 1997; Wendum *et al*, 1997; Rodriguez *et al*, 1999, 2000; Tezcan *et al*, 1999). Even with series that included patients treated with nonanthracycline-containing therapy, only patients treated in the 1970s or patients with a history of cardiac disease were offered routine nonanthracycline-containing therapy (Bartlett *et al*, 1994). No conclusive randomised evidence is available in this uncommon disease entity to test the role of anthracycline-based therapy as first-line treatment. In addition, patient selection, referral pattern and variation in diagnostic criteria between institutions and over the years at the same institution have hampered a clear understanding of the behaviour of this disease.

We investigated whether patients with FL grade 3 treated with anthracycline-based chemotherapy as first-line therapy (A1 group) had a better outcome than those treated with other regimens (non-A1 group). No survival differences were observed between the A1 group and the non-A1 group even when patients with limited stage who received RT alone were excluded. However, no events in OS or FFS occurred after 4 years in the A1 group suggesting that some patients were possibly cured by anthracyclines. This is relatively promising for FL as FL grade 1 and grade 2 had a constant rate of recurrence or progression. Nevertheless, long-term survivors also occurred in the non-A1 group suggesting that a subgroup of patients could be spared from the potential morbidity of anthracyclines, especially the elderly. This is particularly relevant with the introduction of anti-CD-20-targeted therapy such as rituximab, ^131^I-labelled tositumomab and ^90^Y-labelled ibratumomab. Rituximab has shown activity in relapsed FLCL as a single agent (McLaughlin *et al*, 1998). It has also been shown to have superior survival in elderly patients with DLBCL when used in combination with regimen including cyclophosphamide, doxorubicin, vincristine and prednisolone (CHOP) compared with CHOP alone (Coiffier *et al*, 2002). The incorporation of these targeted therapies into combination chemotherapy may obviate the need for anthracyclines in the front-line therapy of FL grade 3. In our series, patients with FL grade 3b did not experience disease relapse or progression after receiving anthracycline-based chemotherapy. Whereas this was a small subgroup analysis and should be interpreted with caution, the trend in favour of better outcome for grade 3b patients may suggest that they represent a biologically distinct group compared with patients with grade 3a, especially when treated with anthracycline-based chemotherapy.

The international prognostic index (IPI) (The International Non-Hodgkin's Lymphoma Prognostic Factors Project, 1993) originally developed for aggressive lymphoma has been shown to correlate with the outcome of patients with FLCL (Bartlett *et al*, 1994; Rodriguez *et al*, 1999, 2000) The international prognostic index could therefore be used to aid clinical decision making. Since not all baseline performance status and laboratory results were available for the present analysis and because some patients were treated nearly 30 years ago and some received initial treatment at another institution, it was decided not to construct a retrospective prognostic model using the available data.

In conclusion, there are long-term survivors among FL grade 3 patients with a continuing risk of relapse. Anthracyclines did not appear to influence survival or disease relapses when given as front-line therapy in our series. Given the caveat that the number of patients with grade 3 (especially grade 3b) in our series was very small, these data are hypothesis-generating. The role of anthracyclines in FL grade 3 patients should therefore be further evaluated in large randomised studies especially with the advent of anti-CD-20-targeted therapy.
